# Giant Cell Tumor of the Distal Ulna With Contiguous Involvement of the Distal Radius: A Report of a Rare Case

**DOI:** 10.7759/cureus.113236

**Published:** 2026-07-23

**Authors:** Pravendra Singh, Aniket Gupta, Harsh Gupta, Milan Kevadiya, Sumit Sanghani

**Affiliations:** 1 Orthopaedics, Maharishi Markandeshwar Institute of Medical Sciences, Ambala, IND

**Keywords:** bone tumor, case report, contiguous extension, distal radius, distal ulna, en bloc resection, giant cell tumor

## Abstract

Giant cell tumor (GCT) of bone is a benign but locally aggressive neoplasm that commonly affects skeletally mature individuals. While GCT most commonly affects the distal femur, proximal tibia, and distal radius, involvement of the distal ulna is uncommon. Contiguous extension of a distal ulna GCT to the adjacent distal radius is exceedingly uncommon and presents unique diagnostic and surgical challenges.

We report a 23-year-old right-hand-dominant male who presented with a four-month history of progressive pain and swelling of the distal forearm. Radiographs demonstrated an eccentric, expansile lytic lesion involving the distal ulna with cortical thinning. Magnetic resonance imaging (MRI) revealed a subarticular lytic lesion with cortical breach and surrounding marrow edema. An image-guided core needle biopsy confirmed GCT. The lesion was classified as Campanacci Grade III. The patient underwent en bloc resection of the distal ulna. Intraoperatively, a small lytic lesion was identified on the adjacent ulnar surface of the distal radius and excised with wide margins. Histopathological examination confirmed identical GCT features in both specimens, establishing contiguous tumor extension rather than a separate primary lesion. At the 12-month follow-up, the patient demonstrated full wrist range of motion, good grip strength, and no evidence of local recurrence.

This case highlights that GCT of the distal ulna can rarely extend contiguously to adjacent bones. Meticulous intraoperative inspection of all adjacent bone surfaces is essential to achieve complete oncological clearance and optimize functional outcomes.

## Introduction

Giant cell tumor (GCT) of bone is a benign, locally aggressive tumor composed of mononuclear stromal cells and osteoclast-like multinucleated giant cells. While GCT most commonly affects the distal femur, proximal tibia, and distal radius, involvement of the distal ulna is uncommon [1,2]. It represents approximately 5% of all primary bone tumors and commonly affects skeletally mature individuals between the second and fourth decades of life [3]. The tumor typically arises in the epiphysis of long bones, with the distal femur, proximal tibia, and distal radius being the most frequently involved sites. Rarely, GCT may occur in the sacrum (4%-9%), pelvis (3%-6%), small bones of the hands and feet (1%-2%), and lumbar vertebrae (<1%) [3,4]. GCTs of the distal ulnar metaphysis are extremely rare, accounting for only 0.45% to 3.2% of all GCT cases, and GCTs crossing interosseous margins account for <1% of all cases.

Although histologically benign, GCT can demonstrate aggressive local behavior and may recur following treatment. Distant metastasis occurs in a small percentage of patients, most commonly involving the lungs. However, contiguous extension to adjacent bones is extremely uncommon.

Due to the rarity of distal ulna GCT and the possibility of unusual local spread, accurate imaging evaluation and complete surgical excision are crucial. We present a rare case of distal ulna GCT with contiguous involvement of the distal radius, managed successfully with en bloc resection.

## Case presentation

**Figure 1:**
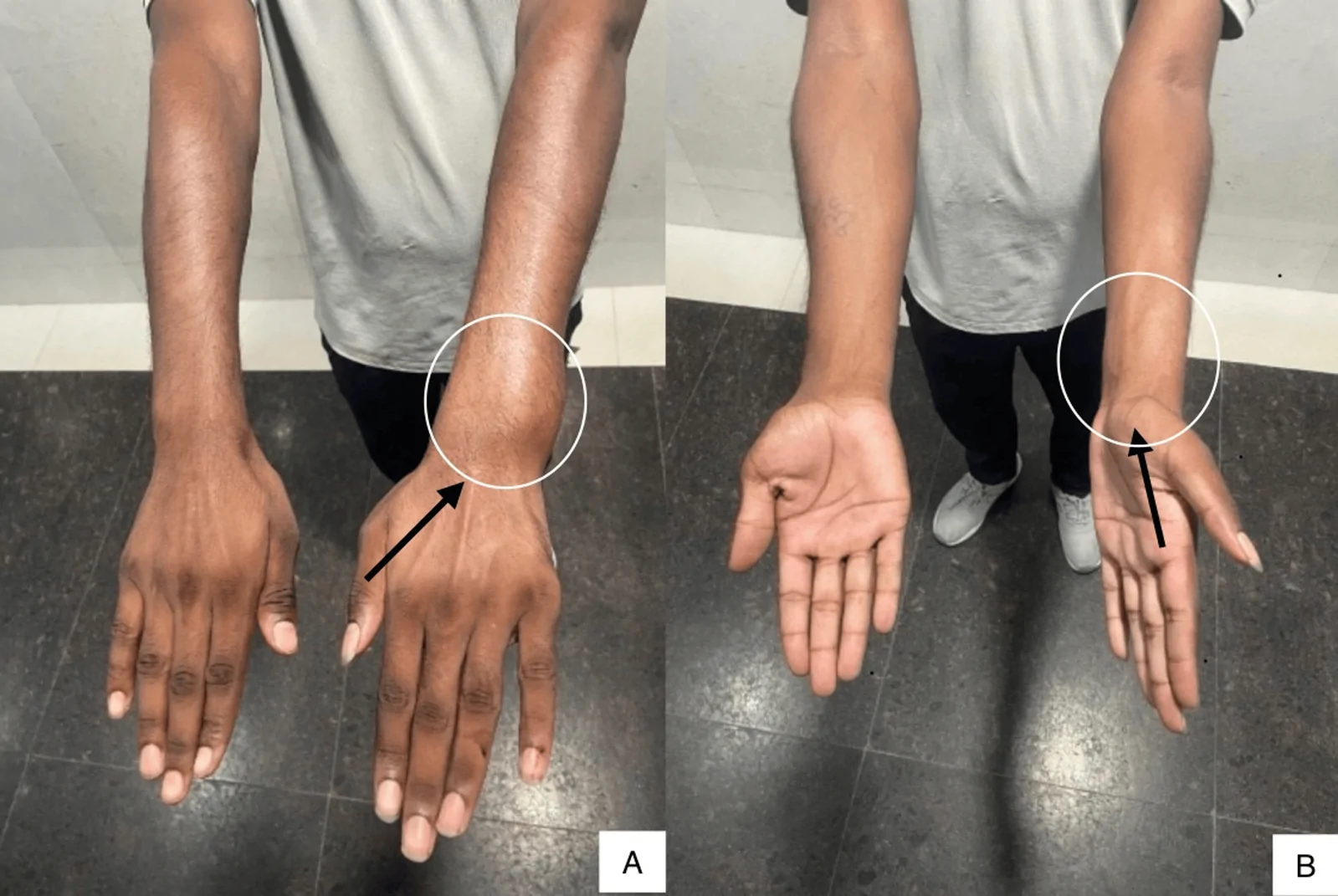
(A-B) Preoperative clinical photograph showing swelling over the distal ulna.

A 23-year-old right-hand-dominant male presented with a four-month history of progressive pain and swelling over the dorsal-medial aspect of the distal forearm. The pain was insidious in onset, gradually progressive, and aggravated by wrist movements. There was no history of trauma, fever, weight loss, or systemic symptoms. A preoperative clinical photograph demonstrating the swelling is shown in Figure 1.

On physical examination, a firm swelling measuring approximately 4 × 3 cm was noted over the distal ulna. The swelling was tender and non-mobile, with normal overlying skin. Wrist movements were mildly restricted due to pain. Grip strength was slightly reduced compared to the contralateral side. Neurovascular examination was normal, with intact radial and ulnar pulses and preserved sensory and motor function of the median, ulnar, and radial nerves.

**Figure 2:**
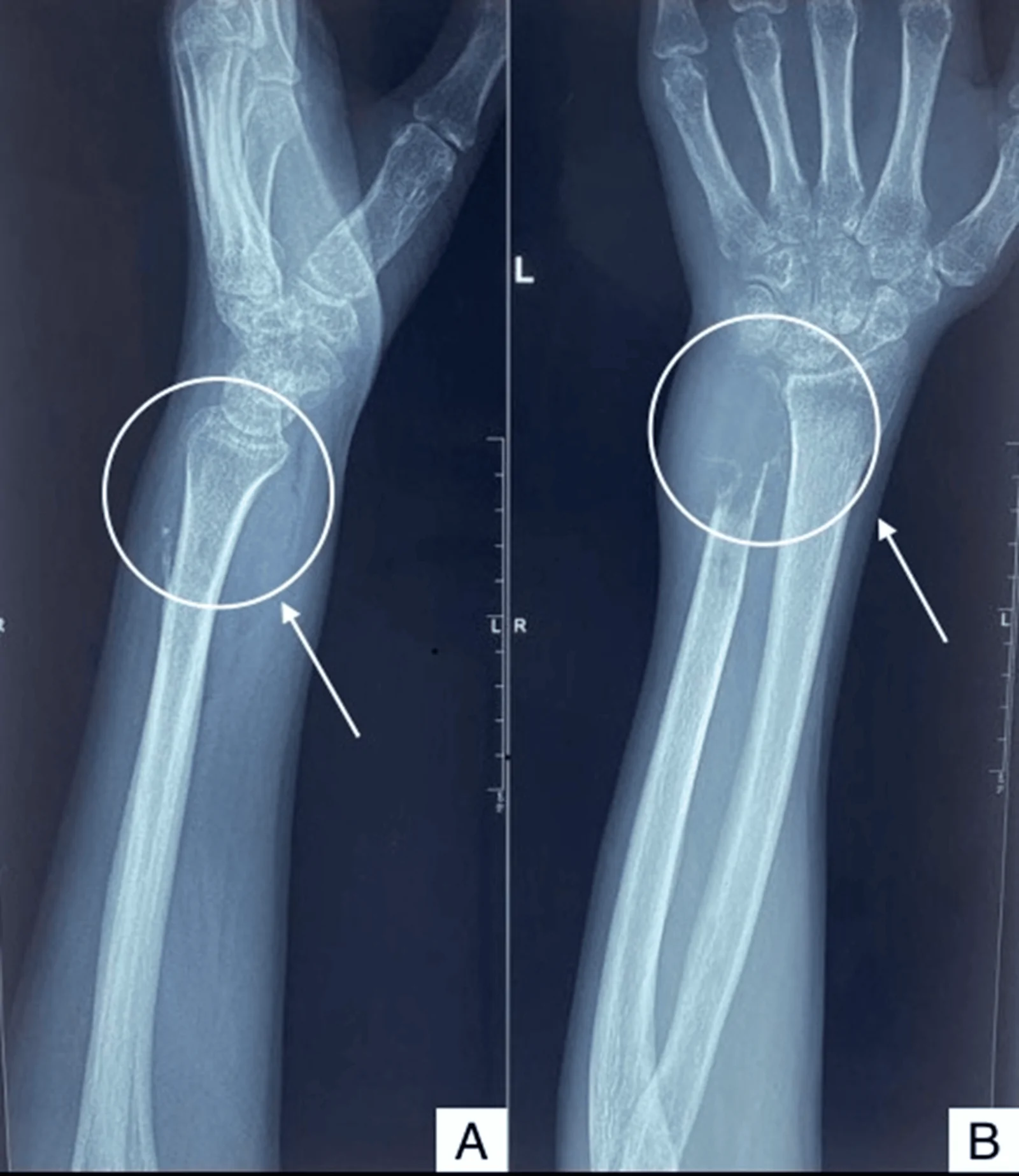
(A-B) Preoperative radiograph demonstrating an eccentric expansile lytic lesion involving the distal ulna.

Plain radiographs of the forearm demonstrated an eccentric, expansile lytic lesion involving the epiphysis and metaphysis of the distal ulna, with cortical destruction and absence of periosteal reaction (Figure 2). The lesion had an ill-defined zone of transition and extended subarticularly. Based on the radiographic features, the tumor was classified as Campanacci Grade III [4].

Magnetic resonance imaging (MRI) revealed a well-defined, eccentric, subarticular lytic lesion involving the distal ulna, with cortical breach and surrounding marrow edema. A small area of soft tissue extension was noted without significant joint involvement.

Considering the aggressive nature of the lesion (Campanacci Grade III) and cortical breach, surgical management with en bloc resection of the distal ulna was planned after obtaining informed consent from the patient [1].

**Figure 3:**
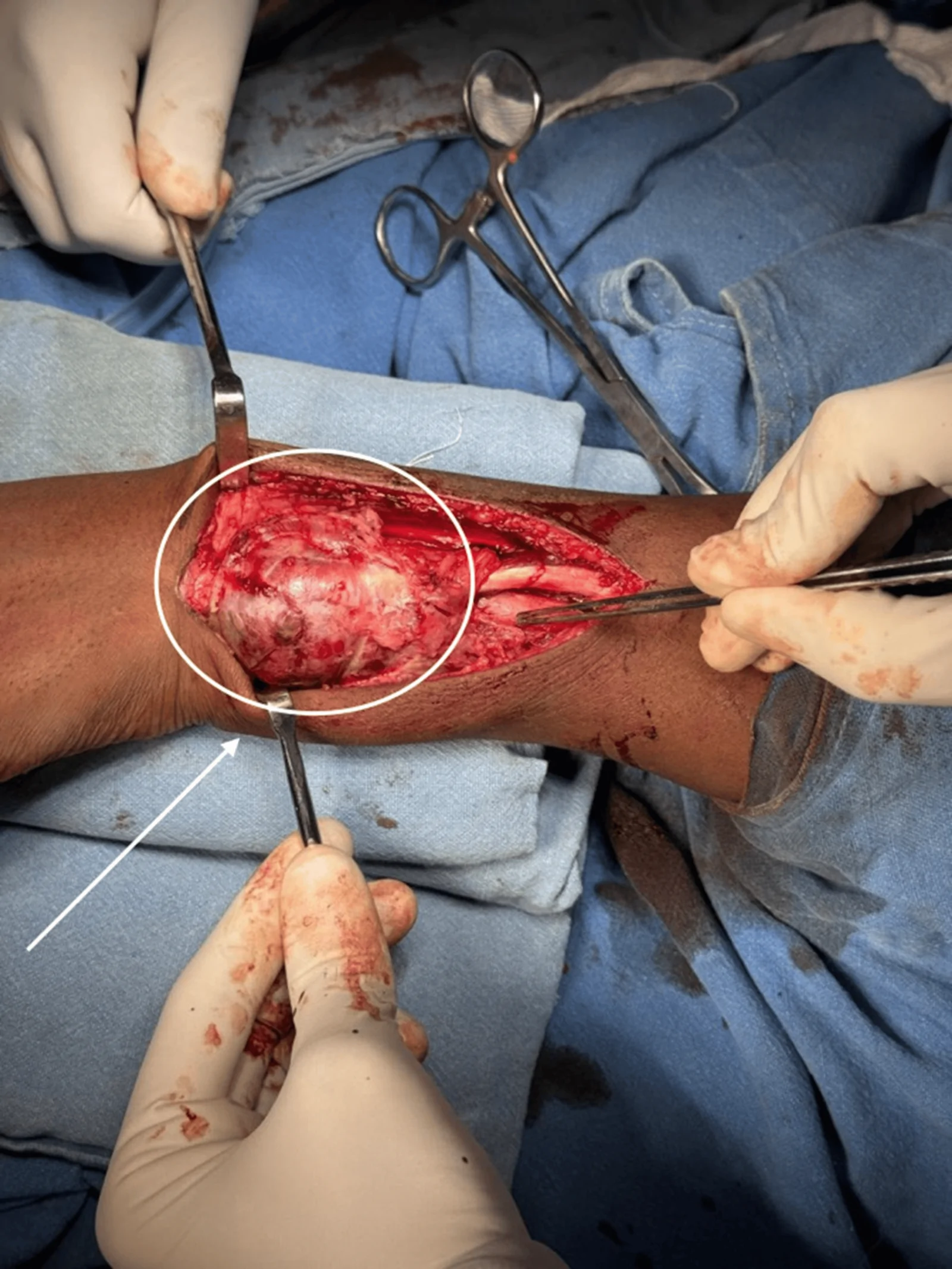
Intraoperative photograph showing en bloc resection of the distal ulna.

**Figure 4:**
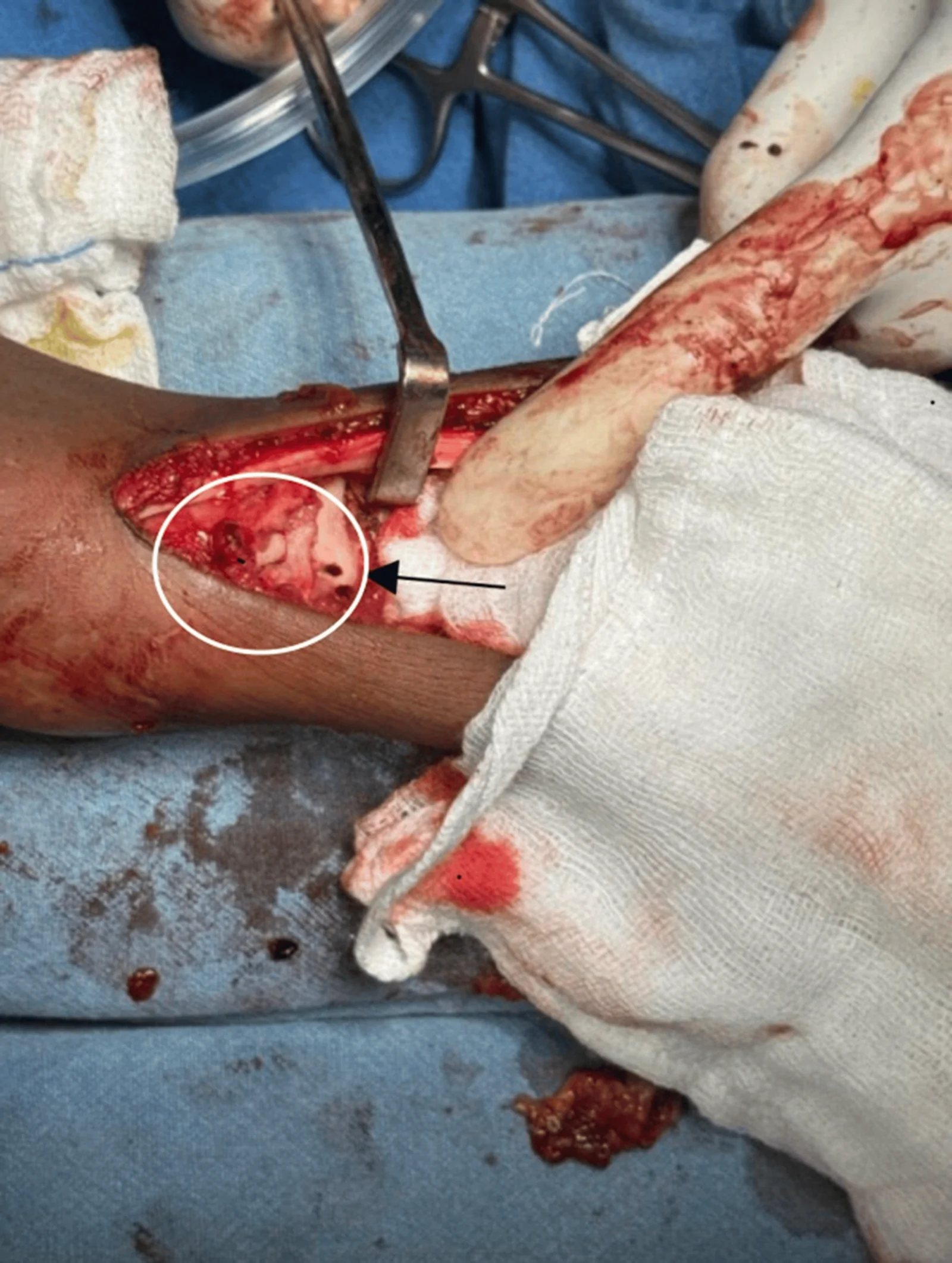
Intraoperative image showing a small lytic lesion on the ulnar aspect of the distal radius.

Under regional anesthesia, a longitudinal incision was made along the subcutaneous border of the distal ulna. Careful soft tissue dissection was performed while preserving the surrounding neurovascular structures. The distal ulna was resected with adequate margins (Figure 3). During intraoperative inspection, a small erosive area was noted on the adjacent ulnar surface of the distal radius (Figure 4). The lesion was excised with wide margins to achieve oncological clearance.

The extensor carpi ulnaris tendon was stabilized to maintain wrist stability. The wound was closed in layers, and a splint was applied.

**Figure 5:**
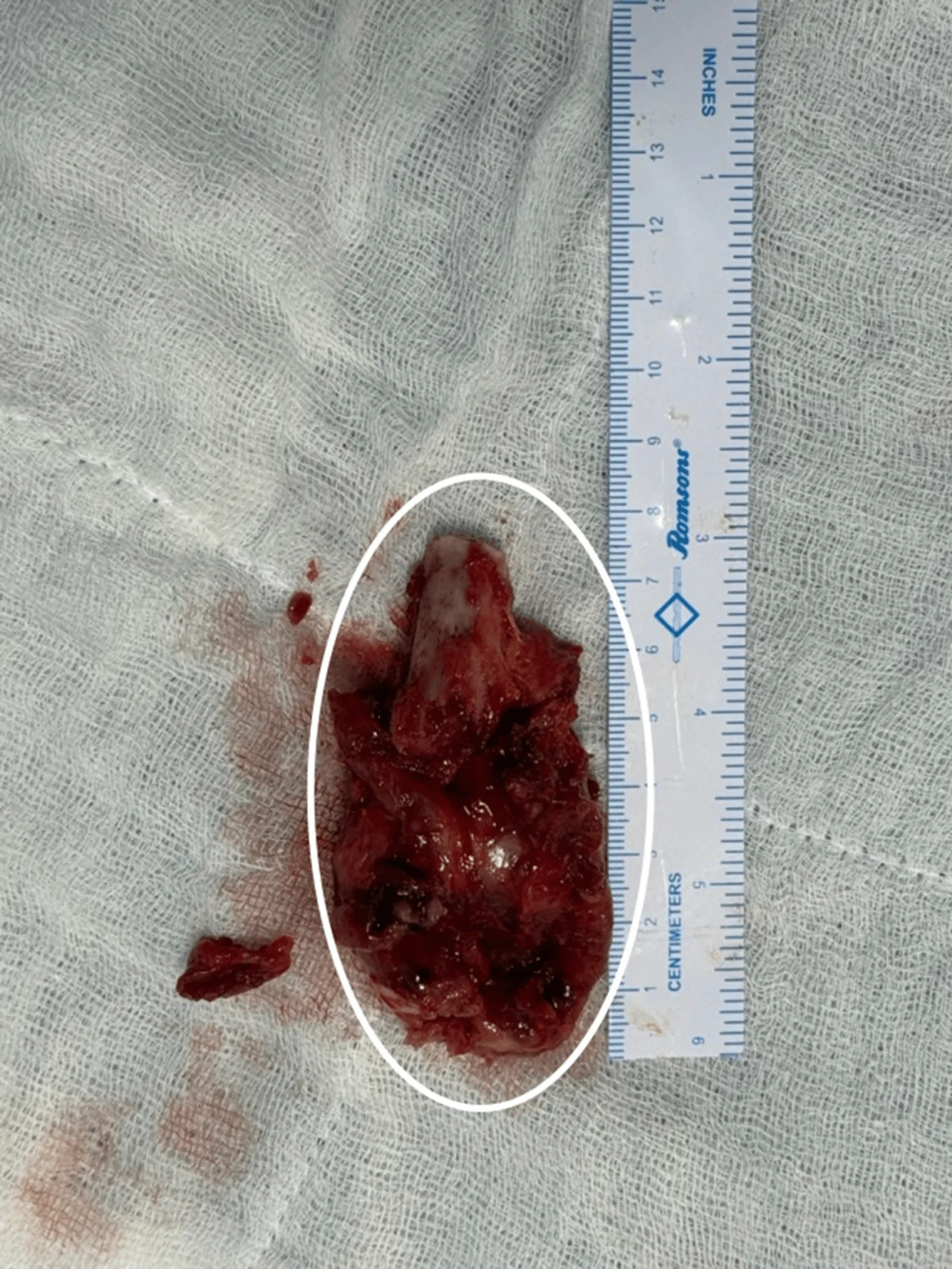
Specimen of the resected distal ulna along with excised lesion from distal radius.

Gross examination of the resected specimen revealed an expansile lytic lesion involving the distal ulna (Figure 5).

**Figure 6:**
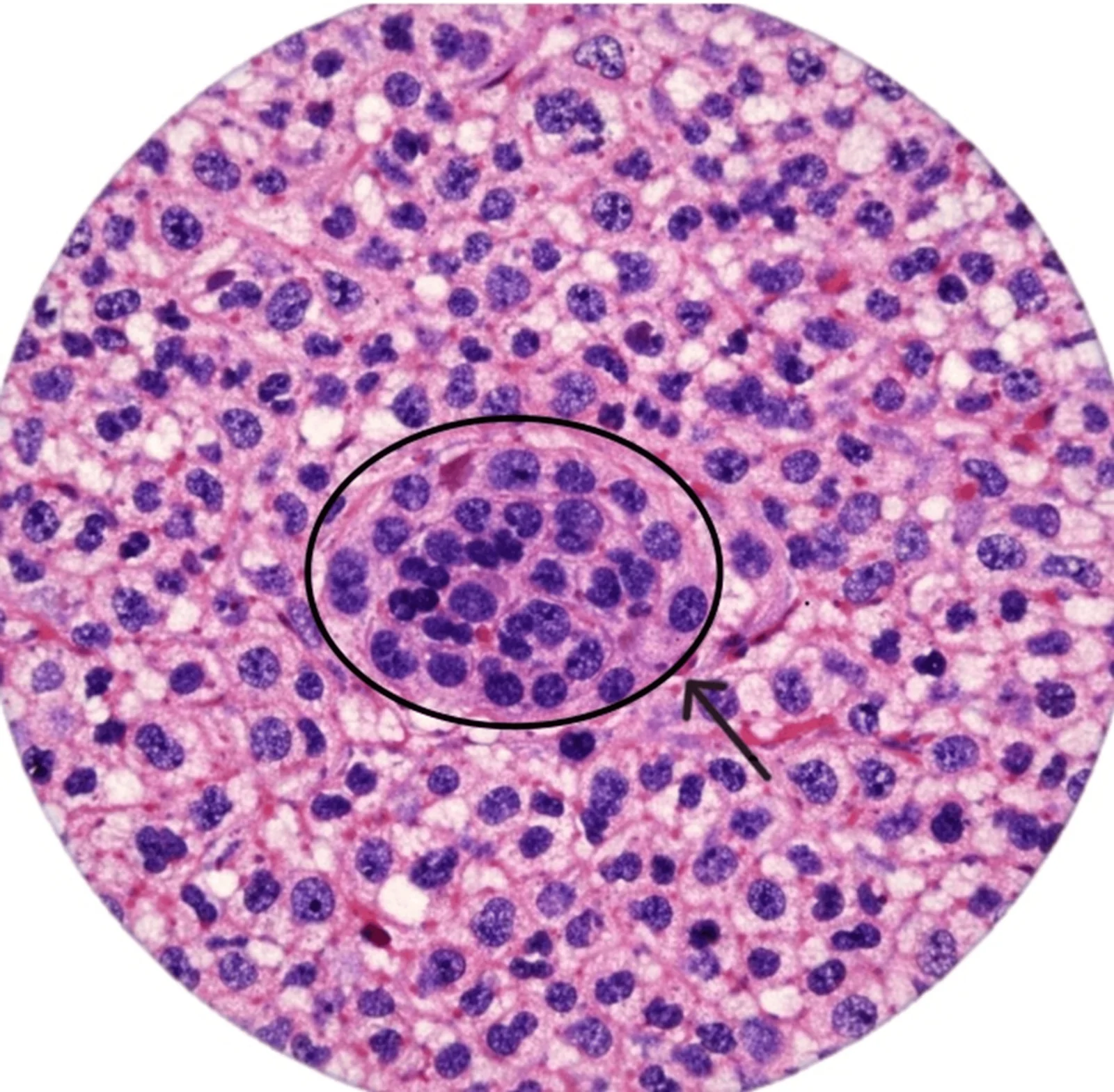
Histopathological slide showing numerous osteoclast-like multinucleated giant cells among stromal cells consistent with giant cell tumor.

Microscopic examination demonstrated numerous osteoclast-type multinucleated giant cells, evenly distributed among mononuclear stromal cells. The stromal cells showed oval- to spindle-shaped nuclei without significant atypia. No features of malignant transformation were noted (Figure 6).

The lesion excised from the distal radius demonstrated identical histopathological features, suggesting contiguous tumor spread rather than a separate primary tumor. This finding is consistent with previously reported rare cases of adjacent bone involvement in GCT [2].

**Figure 7:**
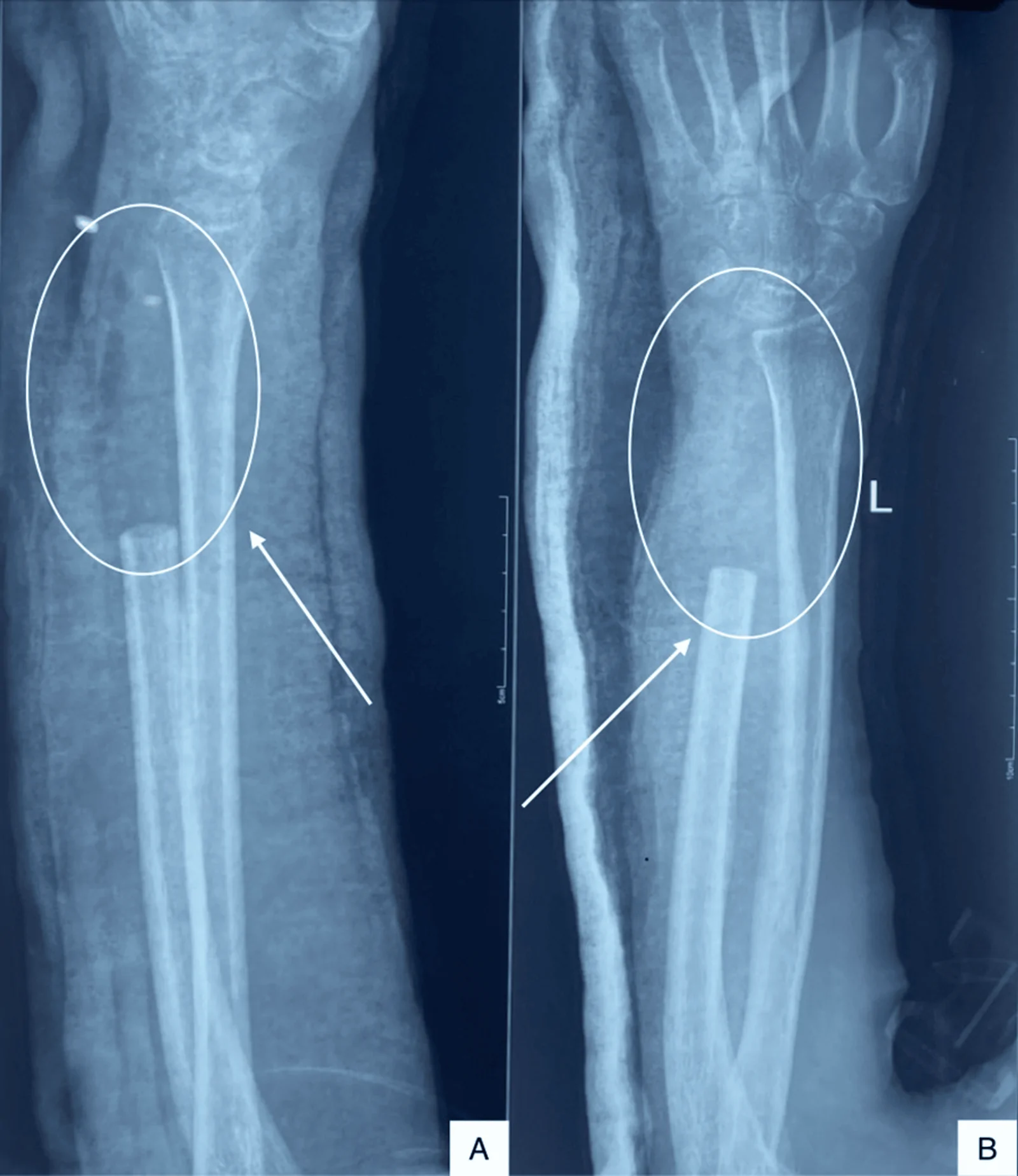
(A-B) Postoperative radiograph showing distal ulna resection with maintained wrist alignment.

The postoperative course was uneventful. Wrist mobilization exercises were started after wound healing. A postoperative radiograph confirming adequate resection with maintained wrist alignment is shown in Figure 7.

At the 12-month follow-up, the patient demonstrated full wrist range of motion, near-normal grip strength compared to the contralateral side, no evidence of local recurrence on radiographs, and no functional limitation in daily activities.

## Discussion

GCT is a benign but locally aggressive bone tumor that primarily affects young adults. The tumor most frequently involves the epiphyseal region of long bones and typically presents with pain, swelling, and limitation of joint movement [3,4].

While GCT most commonly affects the distal femur, proximal tibia, and distal radius, involvement of the distal ulna is uncommon [1,2]. Because of its uncommon location, the diagnosis may be delayed or confused with other lytic lesions, such as aneurysmal bone cyst, chondroblastoma, or giant cell reparative granuloma. Radiographically, GCT typically appears as an eccentric, expansile lytic lesion involving the epiphysis and extending toward the metaphysis, with cortical thinning and absence of periosteal reaction [4]. MRI helps delineate soft tissue extension and intra-articular involvement, guiding surgical planning.

Treatment options for distal ulna GCT include intralesional curettage with adjuvants or wide resection, depending on tumor grade and extent. In distal ulna lesions, en bloc resection is commonly preferred due to the expendable nature of the bone and the desire to minimize recurrence risk [1]. Following resection, the extensor carpi ulnaris tendon can be stabilized to maintain wrist function, as performed in our case.

Contiguous involvement of the distal radius in distal ulna GCT is extremely rare and has been reported in only isolated cases [2]. The present case highlights the importance of careful intraoperative evaluation of all adjacent bone surfaces to identify possible tumor extension and achieve complete oncological clearance.

Adequate surgical margins and long-term follow-up are essential, given the risk of local recurrence associated with GCTs [3].

## Conclusions

GCT of the distal ulna is a rare entity and may occasionally demonstrate unusual patterns of local extension. Contiguous involvement of the distal radius is extremely uncommon and requires careful imaging evaluation and thorough surgical exploration. En bloc resection, with adequate margins, can provide effective tumor control while preserving satisfactory wrist function.
